# Aortic Arch Calcification on Routine Chest Radiography is Strongly and Independently Associated with Non-Dipper Blood Pressure Pattern

**DOI:** 10.5935/abc.20190229

**Published:** 2020-01

**Authors:** Adem Adar, Orhan Onalan, Fahri Cakan, Ertan Akbay, Ekrem Karakaya

**Affiliations:** 1 Karabuk University Faculty of Medicine - Cardiology, Karabuk - Turkey

**Keywords:** Thoracic, Aorta/physiopathology, Calcification, Calcinosis, Cardiomyopathies, Hypertension/imaging diagnosis, Ventricular Function,Left, Antihypertensive Agents/therapeutic use, Blood Pressure Monitoring Ambulatory, Heart Rate

## Abstract

**Background:**

Non-dipper blood pressure (NDBP) is one of the important causes of hypertension-related target organ damage and future cardiovascular events. Currently, there is no practical tool to predict NDBP pattern.

**Objectives:**

The aim of this study was to investigate the relationship between aortic arch calcification (AAC) on chest radiography and NDBP pattern.

**Methods:**

All patients referred for ambulatory BP monitoring test were approached for the study participation. NDBP was defined as the reduction of ≤10% in nighttime systolic BP as compared to the daytime values. AAC was evaluated with chest radiography and inter-observer agreement was analyzed by using kappa statistics. Univariate and multivariate logistic regression analysis was conducted to assess the association of AAC and NDBP pattern. A 2-tailed p-value < 0.05 was considered statistically significant.

**Results:**

A total of 406 patients (median age: 51.3) were included. Of these, 261(64%) had NDBP pattern. Overall, the prevalence of AAC was 230 (57%). Non-dipper group had significantly higher prevalence of AAC (70% vs. 33%, p < 0.0001) as compared to the dipper group. Presence of AAC was a strong and independent predictor of NDBP pattern (OR 3.919, 95%CI 2.39 to 6.42) in multivariate analysis.

**Conclusions:**

Presence of AAC on plain chest radiography is strongly and independently associated with the presence of NDBP pattern.

## Introduction

Hypertension (HT) is the most common cardiovascular disease and it is the leading cause of cardiovascular mortality and morbidity. Blood pressure (BP) follows a circadian pattern with a nocturnal decline of %10 or more as compared with daytime BP. Non-dipper BP (NDBP) pattern is defined as the absence of normal nocturnal decline in BP as compared to daytime measurements. NDBP pattern is associated with disease severity, left ventricular hypertrophy (LVH), proteinuria, secondary forms of HT and insulin resistance.^1-4^ Several forms of HT including NDBP pattern can only be detected by ambulatory BP monitoring (ABPM). Moreover, ABPM is superior to office BP measurements in predicting cardiovascular risk.^5,6^ However, utilization of ABPM to unselected population is not practical and currently, there is no practical tool to predict NDBP pattern.

NDBP pattern has shown to be associated with arterial stiffness.^4,7,8^ Vascular calcification plays an important role in the development of arterial stiffness.^9,10^ Accordingly, aortic arch calcification (AAC) has been shown to be closely related to arterial stiffness.^11,12^ Thus, we hypothesized that AAC on chest radiography predicts NDBP pattern.

## Methods

### Study population

All patients who were referred for ABPM test were approached for the study participation. Indication for ABPM test was left to physician discretion. Following inclusion criteria, we applied: 1) Age ≥ 18-years-old; 2) A valid measurement rate of >85% during the ABPM test. Nighttime workers, patients with inadequate chest x-ray, pregnancy or suspicion of pregnancy, history of moderate to severe cardiac valve disease, malignancy, cardiac or thoracic surgery, coronary artery, cerebrovascular and peripheral vascular disease were excluded from the study. Posterior-anterior (PA) chest radiography and transthoracic echocardiography were performed in all patients. Eligible subjects underwent a comprehensive assessment, including documentation of medical history, physical examination and measurement of laboratory variables. Body mass index was calculated as the weight in kilogram divided by height in square meter. Diabetes was defined as being on treatment with insulin or oral anti-diabetic drugs. HT and hyperlipidemia were defined as the use of anti-hypertensive drugs or lipid-lowering drugs, respectively. The institutional ethics committee approved the study protocol. Patients were divided into two groups according to circadian BP pattern; non-dipper and dipper group.

### Ambulatory blood pressure monitoring

ABPM studies were carried out using a Mobil-O-Graph (M-o-G; I.E.M, Germany) monitoring device. The first hour was discarded from the analysis. BP readings were obtained automatically at the 30-min interval and if >85% of the measurements were valid then it was included in the analyses. Daytime, nighttime and 24-hour BP data and the percentage of the decrease in nighttime systolic BP vs. daytime systolic BP were recorded. The default setting for daytime (07:00 to 23:00) and nighttime (23:00 to 07:00) hours were modified appropriately based on the patient’s feedback. NDBP pattern was defined as the reduction of ≤ 10 % in nighttime systolic BP as compared to the daytime systolic BP.

### Evaluation of AAC

**Figure 1:**
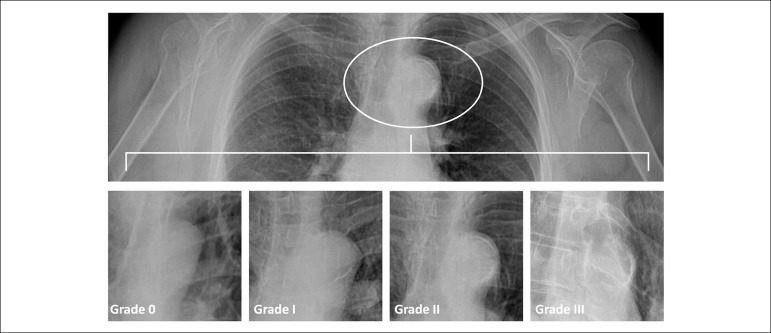
Aortic arch calcification grading.

All patients had chest radiography in the PA view. The standard PA chest radiograph (40 cm×40 cm; Curix HT 1.000G Plus, Agfa, Mortsel, Belgium) was acquired with the patient standing up (Thoramat, Siemens, Erlangen, Germany). The focus-patient distance was 150 cm. An automated exposure control with a fixed tube voltage of 117 kV was used. We noted the presence of calcification in the aortic knob. AAC was graded as follows: Grade 0, no visible calcification; Grade 1, small spots of calcification or thin calcification; Grade 2, one or more areas of thickened calcification, and Grade 3, circular calcification on the aortic knob^13^ (Figure 1). One hundred randomly selected chest radiography for evaluation of AAC were independently evaluated by two cardiologists, who was unaware of the result of the patient’s ABPM data to assess the reliability of AAC diagnosis and Kappa value was detected as 0.812 and p < 0.001.

### Laboratory tests

A venous blood sample was collected from each participant under fasting conditions. Fasting blood glucose, total cholesterol, high-density lipoprotein cholesterol, low-density lipoprotein cholesterol, triglyceride and creatinine were measured by standard laboratory methods. Glomerular filtration rate (GFR) was calculated using CKD-EPI Creatinine Equation.^14^

### Echocardiographic examination

All patients were examined in the left lateral decubitus position using by a commercially available system (Vivid 4 GE Medical System, Horten, Norway) with a phased-array 3.5-MHz transducer. The conventional M-mode and B-mode parameters were measured in accordance with the American Society of Echocardiography guidelines. Left ventricular end-diastolic (LVEDD) and end-systolic (LVESD) diameters, and posterior (PWT) and septal (IVST) wall thicknesses were measured. Left ventricular ejection fraction was measured by using the Teichholz method. Left ventricular mass (LVM) was calculated using the Devereux equation: LVM = 0.8{1.04[([LVEDD + IVST +PWT]^3^ - LVEDD^3^)]} + 0.6.^15^ Left ventricular mass index (LVMI) was calculated by dividing the LVM by body surface area. LVH was defined as LVMI > 115 g/m^2^ for men and 95 g/m^2^ for women.^16^ Based on relative wall thickness (2 x PWT/LVEDD) and the presence or absence of LVH various types of the left ventricular geometrical pattern were defined (normal geometry, concentric LVH, eccentric LVH, and concentric remodeling).

### Statistical analysis

Continuous variables were expressed as mean (standard deviation) or median (interquartile range (IQR)), and categorical variables as number (percentage). The distributions of the continuous variables across the study groups were tested with the Kolmogorov-Smirnov test. Normally distributed data were compared using the Independent Samples t-test and data with non-normal distribution were compared using the Mann-Whitney U test. Categorical data were compared using the chi-square or Fisher’s exact tests when needed.

Univariate and multivariate logistic regression analyses were conducted to assess the association of AAC and NDBP pattern. In multivariate regression analysis (Enter method), the effect size was adjusted for variables with a univariate significance level of < 0.1. Adjusted odds ratios (OR), along with their 95%CIs were presented. A 2-tailed p-value < 0.05 was considered statistically significant. All statistical analyses were performed using the IBM SPSS software (IBM SPSS Statistics for Windows, Version 21.0. Armonk, NY: IBM Corp.)

## Results

A total of 406 patients (mean age 51.3 female 58%) were included. Two hundred sixty-one (64%) had NDBP pattern and classified as non-dipper group. The remaining 145 (36%) patients who had dipper BP pattern were classified as dipper group. As compared to the dipper group, the non-dipper group was older (p < 0.001), had higher LVMI (p = 0.007), prevalence of LVH (p = 0.013), prevalence of HT (p = 0.049) and higher serum triglyceride level (p = 0.013). GFR was significantly lower in the non-dipper group (p < 0.0001). Groups were similar with respect to the remaining baseline characteristics shown in Table 1.

**Table 1:** Baseline characteristics of the study groups

	Non-Dipper (n = 261)	Dipper (n = 145)	p-value
Age (year)	54 (13)	47 (14)	< 0.001
Weight (kg)	80 (13)	79 (13)	0.336
Height (cm)	165 (8)	166 (9)	0.084
Body surface area (m^2^)	1.90 ± 0.18	1.90 ± 0.18	0.864
Body mass index (kg/m^2^)	29 (5)	28 (4)	0.067
Female gender (n, %)	160 (61.3)	76 (52.4)	0.061
Obesity (n, %)	106 (40.6)	51 (35.2)	0.281
Hypertension (n, %)	135 (51.7)	60 (41.4)	0.049
Diabetes (n, %)	59 (22.6)	25 (17.2)	0.201
Hyperlipidemia (n, %)	42 (16.1)	26 (17.9)	0.634
Smoking (n, %)	45 (17.2)	34 (23.4)	0.130
ACE inhibitors (n, %)	71 (27.2)	33 (22.8)	0.326
Angiotensin receptor blockers (n, %)	27 (10.3)	16 (11)	0.829
Calcium channel blockers (n, %)	30 (11.5)	18 (12.4)	0.783
Beta blockers (n, %)	24 (9.2)	16 (11.0)	0.551
Diuretics (n, %)	29 (11.1)	21 (14.5)	0.322
Creatinine (mg/dL)	0.80 (0.20)	0.8 (0.3)	0.910
Glomerular filtration rate (mL/min/1.73 m^2^)	98 ± 13	103 ± 26	< 0.0001
Total cholesterol (mg/dL)	190 ± 39.5	195 ± 39.3	0.200
Triglyceride (mg/dL)	190 (40)	172 (83)	0.013
Low-density lipoprotein (mg/dL)	112 ± 32	114 (33)	0.816
High-density lipoprotein (mg/dL)	47 (11)	46 (12)	0.528
Glucose (mg/dL)	107 (31)	103 (30)	0.088
Left atrial diameter (mm)	35 (4)	34 (4)	0.070
Left ventricular ejection fraction (%)	65 ± 6	64 (5)	0.437
Left ventricular mass index (gr/m^2^)	93 (20)	87 (18)	0.007
Left ventricular hypertrophy (n, %)	61 (23.4)	19 (13.1)	0.013
**Left ventricular geometry (n, %)**			
Normal	49 (18.8%)	30 (20.7%)	0.640
Concentric remodeling	151 (57.9%)	96 (66.2%)	0.099
Eccentric hypertrophy	19 (7.3%)	5 (3.4%)	0.117
Concentric hypertrophy	42 (16.1%)	14 (9.7%)	0.072

Continuous variables are presented as median (interquartile range) or mean (standard deviation); categorical variables are presented as number (percentage). ACE: angiotensin converting enzyme.

There was no difference in daytime DBP, 24-hour SBP and 24-h DBP values between non-dipper and dipper groups. However, daytime SBP was lower in non-dipper groups (p = 0.012). In addition, nighttime SBP (p < 0.0001) and DBP (p < 0.0001) values were significantly higher in non-dipper group (Table 2).

**Table 2:** Ambulatory blood pressure variables of the study groups

	Non-Dipper (n = 261)	Dipper (n = 145)	p-value
Day time mean SBP (mmHg)	124 (14)	127 (13)	0.012
Day time mean DBP (mmHg)	77 (11)	79 (11)	0.407
Nighttime mean SBP (mmHg)	122 (15)	108 (12)	< 0.0001
Nighttime mean DBP (mmHg)	74 (10)	67 (10)	< 0.0001
24-hour mean SBP (mmHg)	124 (14)	121 (12)	0.108
24-hour mean DBP (mmHg)	76 (10)	75 (10)	0.127
Change in SBP (mmHg)	1.9 (7)	19 (5)	< 0.0001
Change in DBP (mmHg)	4 (8)	14 (10)	< 0.0001

Continuous variables are presented as median (interquartile range) or mean (standard deviation).SBP: systolic blood pressure; DBP: diastolic blood pressure

Prevalence of AAC was 57% in our study population. Non-dipper group had significantly higher prevalence of AAC (grade ≥ 1) on chest radiography (p < 0.0001) as compared to the dipper group (Table 3).

**Table 3:** Aortic arch calcification grades in the study groups

	Non-Dipper (n = 261)	Dipper (n = 145)	p-value
**Aortic arch calcification (n, %)**			
Grade 0	79 (30.3)	97 (66.9)	< 0.0001
Grade 1	107 (41.0)	36 (24.8)	
Grade 2	62 (23.8)	11 (7.6)	
Grade 3	13 (5.0)	1 (0.7)	
Aortic arch calcification grade ≥ 1 (n, %)	182 (69.7)	48 (33.1)	< 0.0001
Aortic arch calcification grade ≥ 2 (n, %)	75 (28.7)	12(8.3)	< 0.0001
Aortic arch calcification grade ≥ 3 (n, %)	13 (5.0)	1(0.7)	0.023

Age, body mass index, female gender, HT, GFR, LVMI, presence of LVH, LV geometric pattern of concentric hypertrophy and AAC were associated with the presence of NDBP pattern in univariate logistic regression analysis with a p-value of less than 0.1 (Table 4). Of these; age, body mass index, female gender, HT, GFR, LVMI, presence of LVH and AAC were entered in multivariate regression model. In the multivariate regression analysis, presence of AAC on chest radiography (OR 3.919, 95%CI 2.392 to 6.421) was the only independent predictors of NDBP pattern (Table 5).

**Table 4:** Univariate analysis for non-dipper blood pressure pattern

	β	p-value
Age (year)	0.037	< 0.0001
Body surface area (m^2^)	0.098	0.864
Body mass index (kg/m^2^)	0.049	0.029
Female gender (%)	-0.363	0.082
Obesity (%)	0.231	0.281
Hypertension (%)	0.417	0.046
Diabetes mellitus (%)	0.338	0.202
Hyperlipidemia (%)	-0.130	0.635
Smoking (%)	-0.385	0.131
**Medications (%)**		
Angiotensin converting enzyme inhibitor	0.238	0.326
Angiotensin receptor blocker	-0.072	0.829
Calcium channel blocker	-0.087	0.783
Beta blocker	-0.203	0.552
Diuretics	-0.304	0.323
Creatinine (mg/dL)	-0.241	0.512
Glomerular filtration rate (mL/min/1.73m^2^)	-0.027	0.001
Total cholesterol (mg/dL)	-0.003	0.200
Triglyceride (mg/dL)	-0.002	0.067
Low density lipoprotein (mg/dL)	-0.002	0.608
High density lipoprotein (mg/dL)	0.005	0.587
Glucose (mg/dL)	0.005	0.219
Left atrial diameter (mm)	0.040	0.144
LV ejection fraction (%)	0.009	0.615
LV mass index (gr/m^2^)	0.017	0.003
Left ventricle hypertrophy (%)	0.704	0.014
**LV geometry (%)**		
Normal	-0.121	0.640
Concentric remodeling	-0.356	0.099
Eccentric hypertrophy	0.788	0.125
Concentric hypertrophy	0.585	0.074
Day time mean SBP (mmHg)	-0.015	0.052
Day time mean DBP (mmHg)	-0.009	0.339
Nighttime mean SBP (mmHg)	0.080	< 0.0001
Nighttime mean DBP (mmHg)	0.071	< 0.0001
24-hour mean SBP (mmHg)	0.016	0.050
24-hour mean DBP (mmHg)	0.015	0.130
**Aortic arch calcification (%)**		
Grade 0	Reference category	
Grade 1	1.295	< 0.0001
Grade 2	1.935	< 0.0001
Grade 3	2.770	0.008
Aortic arch calcification grade ≥ 1 (%)	1.538	< 0.0001
Aortic arch calcification grade ≥ 2 (%)	1.497	< 0.0001

SBP: systolic blood pressure; DBP: diastolic blood pressure; LV: left ventricle; β: Regression coefficient.

**Table 5:** Multivariate analysis for non-dipper blood pressure pattern

			95% CI	
	β	OR	Lower	Upper
Age	0.015	1.015	0.988	1.043
Body mass index	0.037	1.038	0.989	1.090
Left ventricular mass index	0.006	1.006	0.992	1.019
Hypertension	0.059	1.061	0.664	1.696
Triglyceride	-0.003	0.997	0.995	1.000
Presence of aortic arch calcification	1.366	3.919	2.392	6.421
Gender	-0.444	0.641	0.405	1.016
Glomerular filtration rate	0.003	1.003	0.979	1.028

CI: confidence interval; OR: odds ratio; β: regression coefficient.

## Discussion

NDBP pattern is one of the important causes of HT-related target organ damage and future cardiovascular events.^1,5,6^ In this study, the presence of AAC on chest radiography was a strong and independent predictor of NDBP pattern.

Diagnosis of HT is generally based on daytime office BP measurements, and nighttime BP and NDBP are usually overlooked in clinical practice. However, the association of nighttime and NDBP with HT-related target organ damage is more powerful than daytime BP.^17-19^ Patients with NDBP pattern are at high risk for target organ damage including myocardial infarction, LVH, carotid artery disease, chronic kidney disease and stroke.^2-4,20^ In the Ohasama study, impaired nocturnal BP decline was associated with cardiovascular mortality.^21^ Each 5% decrease in the decline in nocturnal BP was associated with an approximately 20% greater risk of cardiovascular mortality. Importantly, this association was observed not only in hypertensive, but also in normotensive individuals.^21^ Moreover, cardiovascular mortality and morbidity can be reduced by achieving a better nocturnal BP control.^22^ Thus, effective treatment of HT should include nighttime BP control as well. Currently, ABPM remains the only method for the diagnosis of nocturnal BP variations. Unfortunately, it is relatively expensive, inconvenient for routine usage and not widely available tool. Yet, it may not be practical to perform ABPM in every hypertensive patient. A practical and inexpensive tool may help the filtration of the unselected population for ABPM. Here, in this study, we have shown that AAC on plain chest radiography, an inexpensive and widely available tool, has a strong predictive ability for NDBP pattern.

There are several possible mechanisms that may explain the relationship between AAC and NDBP pattern. First, AAC was found to be strongly correlated with pineal gland calcification which may reduce melatonin secretion during sleep.^23^ Melatonin plays a pivotal role in the regulation of nocturnal BP.^24-26^ Autonomic nervous system activity is involved in the control of the circadian variation of BP^27,28^ and impaired sympathovagal balance with increased sympathetic nervous activity and/or decreased vagal activity has been documented in non-dippers.^29,30^ Melatonin shifts the balance between the sympathetic and parasympathetic system in favor of the parasympathetic system. It may also reduce nighttime BP by its direct arterial vasodilator effect.^25^ Accordingly, exogenous melatonin has been shown to reduce nighttime BP.^31,32^ Thus, a reduced melatonin secretion during nighttime may significantly impair nocturnal BP decline. Second, AAC is closely related with vascular stiffness and loss of arterial compliance^33^ which in turn may impair arterial relaxation capacity. An impaired nocturnal decrease in BP was found to be independently associated with aortic stiffness in patients with nocturnal HT.^34^ Moreover, it has been found that increased arterial stiffness is more associated with nighttime BP load than day time BP.^35^ Third, the relationship between arterial BP and arterial calcifications is likely a bidirectional phenomenon. Increased arterial BP load may facilitate arterial calcification and vice versa. Non-dippers are exposed to an abnormal the nocturnal BP load which may accelerate arterial calcification and stiffness. Fourth, the underlying clinical profile of the patients with impaired nocturnal BP decline and arterial calcification are similar. Both conditions are associated with age, renal diseases, diabetes, sleep apnea, autonomic dysfunction, malignant HT and coronary artery disease.^36,37^

NDBP pattern is associated with disease severity and a higher risk of subsequent cardiovascular events. This risk can be reduced by achieving better dipping patterns and nocturnal BP levels. In clinical practice, many patients with controlled daytime BP levels are not evaluated for the nighttime BP levels. Our results may help to improve detection of NDBP pattern and nocturnal HT. Appropriate treatment of these patients by changing antihypertensive medications or administering the antihypertensive medications in the night may eventually help to improve dipping pattern and patient outcomes.

Both AAC and NDBP are associated with several HT-related target organ damage and future cardiovascular events in patients with HT.^1,5,6,38-40^ In this study, we showed that there is also a strong association between AAC and NDBP. Further studies are needed to confirm our findings and to evaluate the potential association of AAC with other hypertensive subforms.

### Study limitations

This study has several limitations. The small sample size is the main limitation. Our definition of NDBP pattern was based on systolic BP variations. Although this is the most commonly used definition of NDBP, diastolic BP values may also be used to assess NDBP. We did not study autonomic nervous system activity or vascular stiffness parameters to explain the potential mechanistic link between AAC and NDBP. Finally, we did not study the association of AAC with cardiovascular events.

## Conclusion

Presence of AAC on plain chest radiography is strongly and independently associated with the presence of NDBP pattern. Routine use of this simple and inexpensive tool in clinical practice may have additional benefits in the detection and control of the nocturnal BP. Moreover, this tool may help to use of ABPM devices more precisely, which may reduce healthcare cost.
